# Nano-fiber/net artificial bionic dura mater promotes neural stem cell differentiating by time sequence external-oral administration to repair spinal cord injury

**DOI:** 10.7150/thno.102584

**Published:** 2025-01-27

**Authors:** Dapeng Zhang, Wenzhao Wang, Shuwei Han, Huiquan Duan, Mengfan Hou, Xiaolong Zhou, Xianzheng Guo, Haosheng Chen, Xiaohong Kong, Xingshuang Zhang, Hengxing Zhou, Shiqing Feng

**Affiliations:** 1 Department of Orthopedics, Qilu Hospital of Shandong University, Shandong University Centre for Orthopedics, Cheeloo College of Medicine, Shandong University, Jinan 250012, P. R. China.; 2 Advanced Medical Research Institute, Shandong University, Jinan 250012, P. R. China.; 3 The Second Hospital of Shandong University, Cheeloo College of Medicine, Shandong University, Jinan 250033, P. R. China.; 4 Department of Orthopedics, Tianjin Medical University General Hospital, International Science and Technology Cooperation Base of Spinal Cord Injury, Tianjin Key Laboratory of Spine and Spinal Cord, Tianjin Medical University, Tianjin 300052, P.R. China.; 5 Advanced Materials Institute, Qilu University of Technology (Shandong Academy of Sciences), Jinan 250014, P. R. China.; 6 Center for Reproductive Medicine, Shandong University, Jinan, Shandong 250012, P.R. China.

**Keywords:** Spinal cord injury, coaxial electrospinning, artificial bionic dura mater, neural stem cell, nano-fiber/net

## Abstract

**Rationale:** The inflammatory microenvironment and resulting neuronal loss following spinal cord injury (SCI) impede the repair process. Endogenous neural stem cells (NSCs), which are constrained by neuroinflammation and slow differentiation rates, are unable to effectively facilitate recovery.

**Method:** In this study, coaxial electrospinning technology was employed to fabricate a nano-fiber/net artificial bionic dura mater (NABDM). This structure featured a core-shell structure, with nafamostat mesylate (NM) encapsulated in the outer layer of poly (lactic-co-glycolic acid) (PLGA) and neurotrophins-3 (NT3) encapsulated in the inner layer of poly (l-lactic acid) (PLLA).

**Results:** The NABDM modifies the polarization direction of microglia *in vitro*, and promotes the differentiation of NSCs by activating the cGMP-PKG and cAMP signaling pathways. In a mouse SCI model, NABDM effectively reduces local neuroinflammation, accelerates the differentiation of endogenous NSCs, increases the number of mature neurons, and enhances motor, sensory, and autonomic nerve functions in mice.

**Conclusion:** NABDM promotes the differentiation of NSCs and facilitates the repair of SCI in a time sequence external-oral way. This approach represents a rapid and effective new treatment method.

## Introduction

Spinal cord injury (SCI) is a significant condition affecting the central nervous system, imposing substantial burdens on individuals and society, and effective treatments are currently lacking 1. Preliminary studies have indicated that the primary pathophysiological changes associated with SCI include neuron loss and microenvironmental imbalance 2, 3. Consequently, strategies to replenish neuron populations and enhance the neuroinflammatory environment are crucial therapeutic directions. Neural stem cells (NSCs), which can directly differentiate into neurons, serve as essential seed cells. After SCI, endogenous NSCs are activated and recruited to the site of injury. However, their reparative capacity is hindered by the inflammatory environment and slow differentiation rates 4, 5. Addressing these limitations is imperative for effective SCI treatment.

Nafamostat mesylate (nafamostat, NM) is a nonantigenic synthesis inhibitor of trypsin-like serine protease that was approved by the United States FDA for the treatment of acute pancreatitis, shock, and other conditions, and it has good safety profiles 6. In recent years, increasing attention has been given to the anti-inflammatory and neuroprotective properties of NM. Our previous results indicated that NM can protect the blood-spinal barrier, inhibit apoptosis following SCI in rats, and reduce pyroptosis after SCI in mice 7, 8. However, the effect of NM on microglia remains unclear and requires further investigation. Neurotrophin-3 (NT3) is a key member of the classical neurotrophic factor family and plays a crucial role in regulating neuronal development and synaptic function 9-11. However, its expression in the adult central nervous system is significantly diminished 12. Studies have shown that NT3 can exert neuroprotective effects, recruit endogenous NSCs, promote neurogenesis, and enhance synaptic plasticity 13, 14. Despite these benefits, the consistent and stable delivery of these two drugs to the site of SCI remains a challenge, necessitating the development of effective drug carriers.

Biomaterials, including nanomaterials, hydrogels, 3D printing and other technologies, which can play roles in drug delivery and cell scaffold formation and are a new strategy for SCI repair, have played an increasingly important role in SCI research 15-17. Coaxial electrospinning technology is employed to prepare nanofibers with core-shell structures through the electrospinning process. This method enables the synthesis of fiber membranes with diverse morphologies and applications, uses two or more polymer solutions, and is increasingly important in biomedical fields such as drug delivery and antibacterial applications 18, 19. The primary polymers used include polylactic acid (PLA), poly (lactic-co-glycolic acid) (PLGA), and poly (l-lactic acid) (PLLA). PLGA is a synthetic hydrophobic polyester composed of lactic acid and glycolic acid monomers. It has excellent biocompatibility, biodegradability, biosafety, and versatility and is FDA approved. Therefore, it is a crucial choice for drug delivery system carriers 20, 21. Similarly, PLLA is an FDA-approved polymer for human clinical use and is known for its good biodegradability and biocompatibility. It is widely used in the production of biomaterials and serves as an effective drug carrier platform 22-24. A nano-fiber/net is a structure with cobweb fibers distributed among the backbone of a nanofiber 25. It is commonly used in air filtration and sensor research, and an increasing number of researchers are applying these structures in biomedicine, including slow drug release and skin healing 26, 27.

In this study, PLLA was used as a carrier for NT3, and PLGA was used as a carrier for NM to prepare core-shell nano-fiber/net using coaxial electrospinning technology. PLGA loaded with NM was positioned in the outer layer to reduce inflammation during the acute phase following SCI, modify the polarization direction of microglia, and create an optimal differentiation environment for NSCs, which serve an external function. Once the sustained release of NM from the outer layer was complete, the release of the PLLA loaded with NT3 from the inner layer began, which was then taken up by endogenous NSCs orally, promoting their differentiation and achieving sequential drug delivery. However, the hydrophobic properties of both PLGA and PLLA could affect the wettability of the nano-fiber/net membrane, leading to suboptimal drug release. To address this, polyethylene glycol (PEG) was added to the outer layer of PLGA to enhance hydrophilicity, ensuring that the membrane fully contacted body fluids. This modification resulted in a nano-fiber/net artificial bionic dura mater (NABDM) with mechanical properties similar to those of natural dura mater, offering both support and stable drug release. This innovative approach provides a new method for the treatment of SCI.

## Results

### Preparation of NABDM

**Figure 2:**
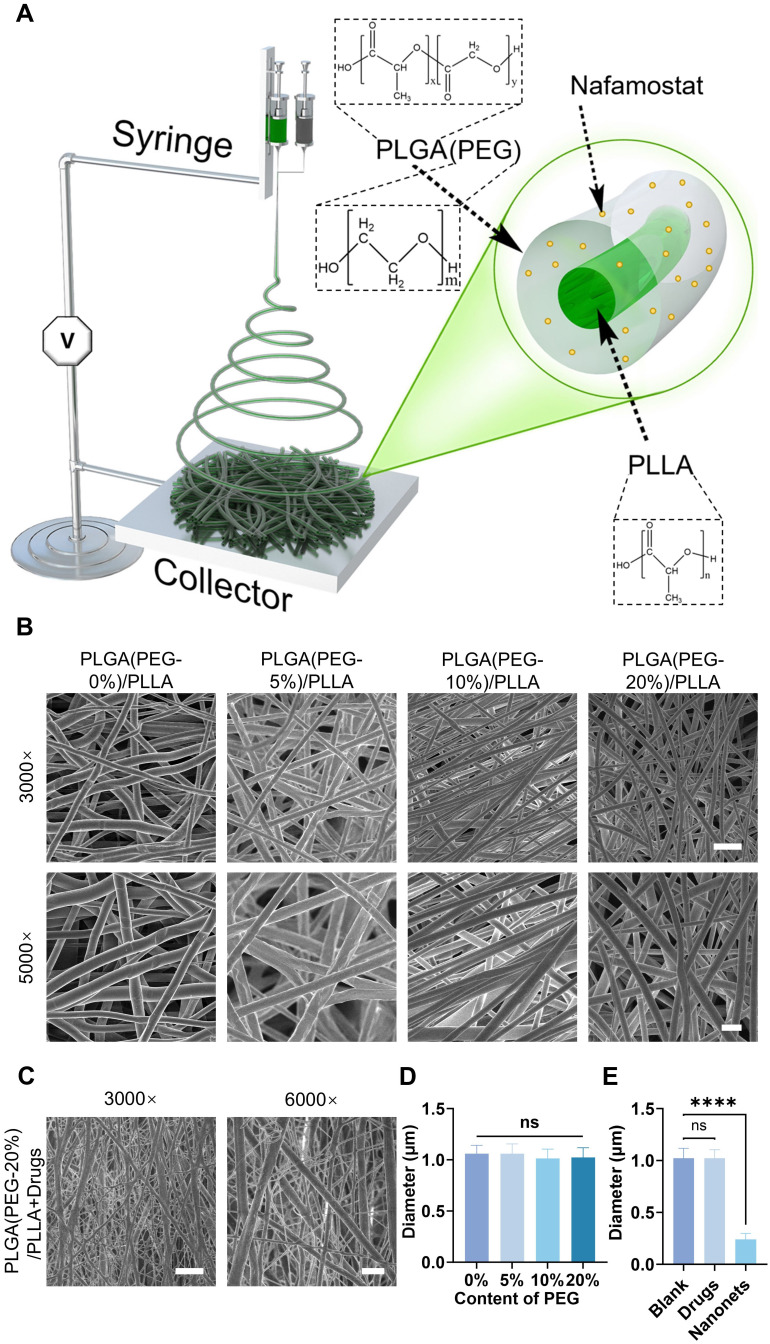
Synthesis of coaxial electrospinning NABDM. A) The diagram of establishing coaxial electrospinning NABDM. B) The SEM of PLGA/PLLA membrane with different content of PEG in PLGA. The scar bar of 3000× is 5 μm, and 5000× is 2 μm. C) The SEM of PLGA(PEG-20%) /PLLA membrane with NM in PLGA and NT3 in PLLA. The scar bar of 3000× is 5 μm, and 6000× is 2 μm. D and E) The analysis of diameter in SEM images. n=3, ****p < 0.0001, ns, not significant.

Using coaxial electrospinning technology, a coaxial nozzle was connected to two independent syringes: one containing PLGA solution with NM and the other containing PLLA solution with NT3. The resulting artificial bionic dura mater was collected on aluminum foil (Figure 2A). Scanning electron microscopy (SEM) images revealed the morphology of the artificial bionic dura mater, with an average nanofiber diameter of 1 μm. Comparisons of fiber membranes with varying concentrations of PEG revealed minimal differences in nanofiber diameter across concentrations, indicating good stability (Figure 2B, 2D, and S1). Upon the addition of NM and NT3, nanonets appeared between the nanofibers, forming NABDM (Figure 2C, 2E, and S2A). This phenomenon may be attributed to changes in the precursor concentration and intermolecular forces due to drug addition, leading to the formation of nanonets.

### Characteristics of the NABDM

**Figure 3:**
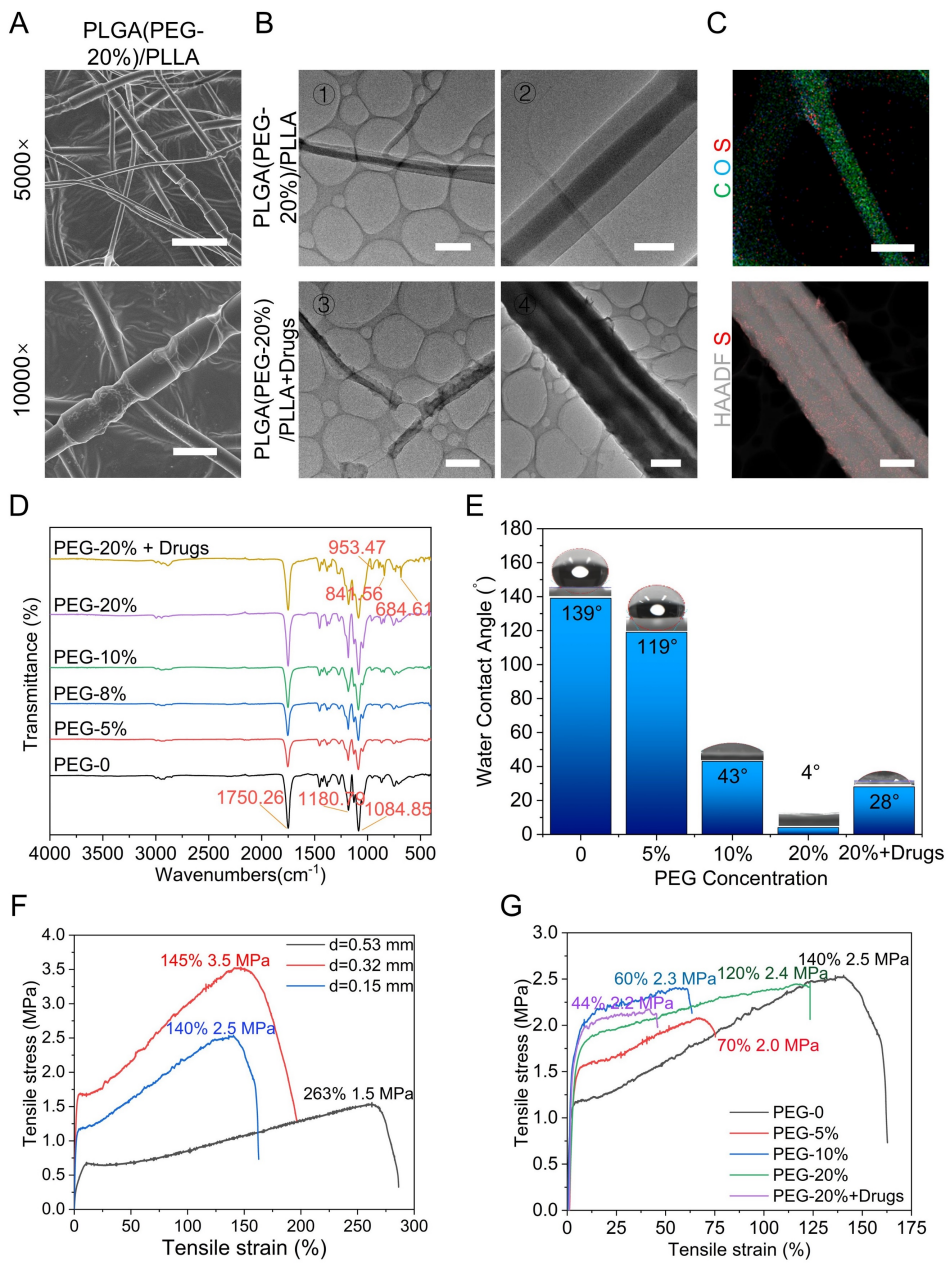
Characterizations of coaxial electrospinning NABDM. A) The SEM of PLGA(PEG-20%)/PLLA core-shell structure membrane. The scar bar of 5000× is 5 μm and 10000× is 2 μm. B) The TEM of PLGA(PEG-20%)/PLLA membrane with or without drugs. The scar bar of ①③④ is 2 μm, and ② is 500 nm. C) The EDS of PLGA(PEG-20%) /PLLA+drug. The scar bar of C O S is 500 nm, and HAADF S is 2 μm. D) The FTIR of different membrane. E) The hydrophilic test of different membrane. F) The mechanical test of membrane with different thickness. G) The mechanical test of membrane with different ingredient.

To demonstrate the successful preparation of core-shell nanofibers, we modified the preparation procedure by replacing the continuous inflow of the outer shell liquid with an intermittent inflow. This adjustment was made to observe changes in the nanofiber structure. SEM results revealed a distinctive bamboo-like structure resulting from this modification, thus confirming the formation of the core-shell structure (Figure 3A and S2B). Transmission electron microscopy (TEM) images further confirmed the presence of a core-shell double-layer structure with distinct light and dark regions. Compared with that in the control group, the smoothness of the shell significantly changed after drug incorporation (Figure 3B). Energy-dispersive spectroscopy of the PLGA (PEG-20%)/PLLA+drug membrane indicated that it was primarily composed of carbon (C) and oxygen (O), with sulfur (S) from the NM added to the shell structure evenly distributed on the nanofiber surface (Figure 3C). These results confirmed the successful construction and drug loading of the core-shell nanofibers. Fourier transform infrared spectroscopy (FTIR) was used to analyse the chemical composition of the fiber membranes. The results revealed that the peaks corresponding to different PEG concentrations were consistent, indicating that the introduction of PEG did not alter the chemical structure of the PLGA/PLLA framework. The characteristic peaks at 953.47, 841.56, and 684.61 cm^-1^ were attributed to the added drugs, confirming their successful incorporation (Figure 3D). To assess wettability, hydrophilicity tests were conducted to determine the interaction of the fiber membrane with body fluids. The results revealed increased hydrophilicity with increasing PEG concentration. Although the hydrophilicity of the drug-loaded group was not as high as that of the 20% PEG group, it exceeded that of the 10% PEG group, ensuring efficient drug release (Figure 3E, S3-4). The mechanical properties were evaluated using a universal testing machine. Initial tests on the PLGA/PLLA group revealed that thicker samples exhibited better tensile strain, with a maximum tensile strain of 263% observed at a thickness of 0.53 mm. The tensile stress ranged from 1.5 to 3.5 MPa, which is sufficient for SCI framework applications (Figure 3F). Mechanical tests on groups with varying PEG concentrations revealed minimal changes in the mechanical properties when the PEG concentration exceeded 10%. Drug addition slightly reduced the mechanical properties (Figure 3G). SEM results indicated that the mechanical properties of NABDM decreased due to nanonets formation, but the tensile strain increased, enhancing the tensile resistance. Finally, drug release was demonstrated by detecting the drug concentration, and material degradation was tested by hydrolysis *in vitro* and subcutaneous degradation, confirming the drug release and degradation of PLGA and PLLA (Figure S5-6).

### NABDM inhibits neuroinflammation and promotes NSC differentiation *in vitro*

**Figure 4:**
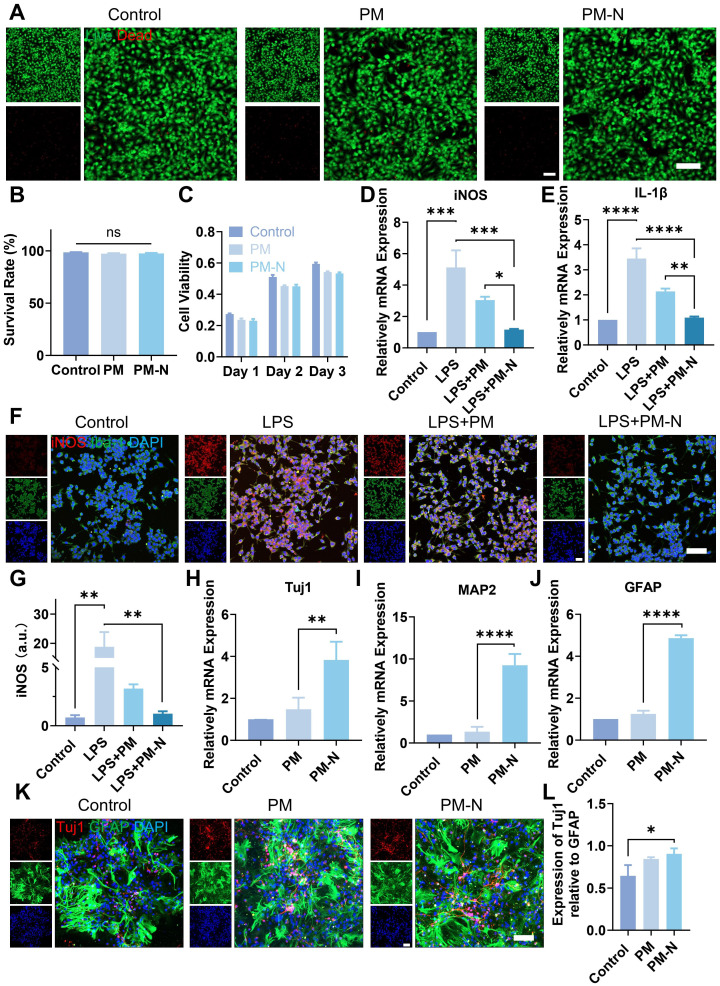
Effect of NABDM on NSCs and BV2 cells. A) Live/dead staining images of NSCs cultured with different scaffolds. B) Statistical analysis of the cell survival rate. ns, not significant. C) Cell viability of NSCs cultured with different scaffolds. D, E) The mRNA expression of iNOS and IL-1β analyzed by RT-qPCR cultured for 1 days, *p < 0.05, **p < 0.01, ***p < 0.001, ****p < 0.0001. F, G) Immunofluorescent staining and statistical analysis of the average fluorescence intensity of iNOS and Iba-1 after 1 days of culture, **p < 0.01. H-J) The mRNA expression of Tuj1, MAP2 and GFAP analyzed by RT-qPCR cultured for 7 days, **p < 0.01, ****p < 0.0001. K, L) Immunofluorescent staining and statistical analysis of the average fluorescence intensity of Tuj1 and GFAP after 7 days of culture, *p < 0.05.

Next, the biocompatibility of NABDM with NSCs was evaluated using live/dead staining and CCK-8 assays. First, the extracted cells were identified as NSCs (Figure S7). As shown in Figure 4A-B, there was little difference in the survival rates of cells cultured on the PM(PLGA(PEG-20%)/PLLA membrane) and PM-N(NABDM) groups compared with the control group. Additionally, the results of the CCK-8 assay were consistent with those of the live/dead staining (Figure 4C). With increasing culture time, the cells in all the groups tended to proliferate well. The numbers of cells in the PM and PM-N groups were slightly lower than that in the control group at different time intervals, likely due to the fiber structure of the membrane. Thus, these results demonstrate that NABDM prepared using coaxial electrospinning technology has excellent biocompatibility.

After SCI, microglia, which are innate immune cells in the central nervous system, become activated and accumulate at the injury site. M1 microglia release proinflammatory factors such as interleukin-1β (IL-1β) and inducible nitric oxide synthase (iNOS), exacerbating damage. This inhibitory inflammatory environment is not conducive to nerve regeneration post-SCI. Therefore, we investigated whether NABDM could alter the polarization state of BV2 cells (a microglial cell line) to improve the inhibitory inflammatory environment using real-time quantitative polymerase chain reaction (RT-qPCR) to assess the mRNA levels. Lipopolysaccharide (LPS) was used to induce M1 polarization in BV2 cells, simulating the inhibitory inflammatory environment. As shown in Figure 4D-E, compared with the control, LPS promoted the expression of the M1 phenotype markers IL-1β and iNOS. After incubation with the fiber membrane for one day, the expression levels of IL-1β and iNOS significantly decreased, particularly in the PM-N group. Immunofluorescence staining was used to further confirm the anti-inflammatory effect at the protein level. Iba-1 was used as a marker for BV2 cell localization, and the fluorescence intensity of iNOS was used to visualize M1 phenotype expression. As shown in Figure 4F-G, after one day of incubation with the fiber membrane, the fluorescence intensity of iNOS significantly decreased, especially in the PM-N group. Therefore, the above results indicate that NABDM can modify the polarization state of BV2 cells, through the effect of NM, thereby improving the inhibitory inflammatory environment to support nerve regeneration.

The effectiveness of recovery following SCI depends significantly on the reconstruction of neural networks. In addition to its anti-inflammatory effects, NABDM was designed to regulate endogenous NSC differentiation and promote neural network reconstruction in the damaged spinal cord. After a 7-day incubation with the membrane, RT-qPCR analysis (Figure 4H-J) was conducted to detect neural-related gene expression at the mRNA level. Compared with that in the control group, the expression of beta-tubulin III (Tuj1), a marker for immature and mature neurons, was increased 3.8-fold in the PM-N group. The expression of microtubule-associated protein 2 (MAP2) increased by 9.2-fold, and the expression of glial fibrillary acidic protein (GFAP), a marker for astrocytes, increased by 4.9-fold in the PM-N group compared with the control group. These RT-qPCR results indicate that NABDM accelerated the neural differentiation of NSCs. Immunofluorescence staining further confirmed the neural differentiation effect at the protein level. As shown in Figure 4K-L, the fluorescence intensities of Tuj1 and GFAP were greater in the PM-N group than in the control group. Importantly, the relative expression of Tuj1 to GFAP suggested an increased proportion of neurons following incubation with the fiber membrane. Thus, neural differentiation assays demonstrated that NABDM accelerates neural differentiation, promotes neuronal maturation, and increases the proportion of neurons, due to the incorporation of NT3 in NABDM.

### Mechanism of NABDM promote neural differentiation

**Figure 5:**
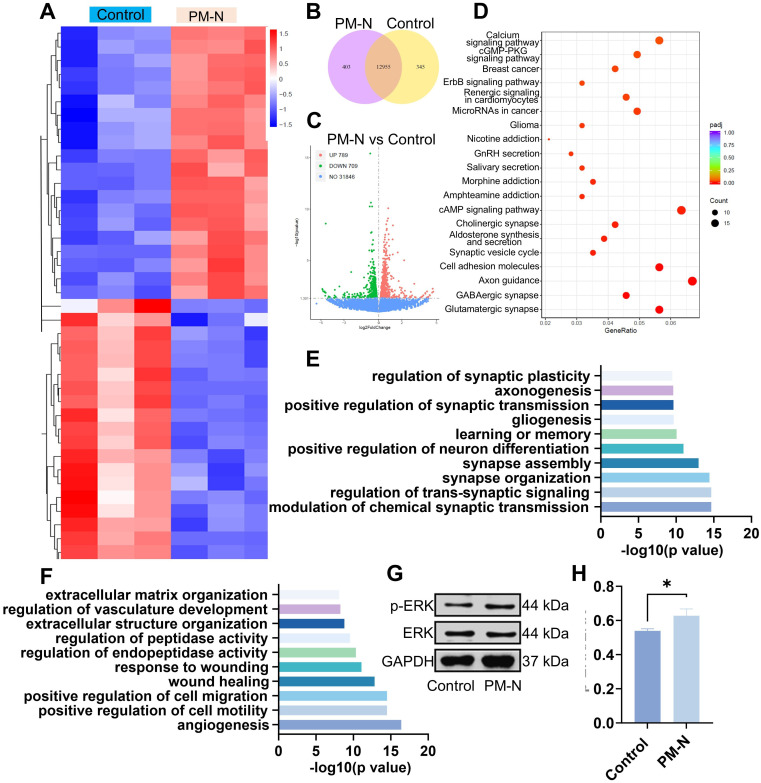
Mechanism underlying NSC differentiation based on RNA sequencing. A) Heat map showing the differentially expressed genes between NSCs cultured on control group and NSCs cultured on PM-N group. B) Venn diagram displaying the differentially expressed genes for pairwise comparison between control after 7 days of culture and PM-N after 7 days of culture. C) Volcanic maps for differentially expressed genes. Red dots, significantly upregulated genes. Green dots, significantly downregulated genes. Blue dots, no differentially expressed genes. D) The differentially expressed genes were analyzed separately by KEGG enrichment analysis. The top 20 enriched pathways (p < 0.05) were presented. E, F) All differentially expressed genes were classified into one or more biological processes (BPs). Significantly upregulated or downregulated BPs were presented. G, H) WB analysis of the proteins associated with signaling pathways cultured for 7 days. n=3, *p < 0.05.

RNA sequencing analysis was conducted to investigate the mechanisms by which NABDM promotes the neural differentiation of NSCs. The heatmaps in Figure 5A display the top 40 significantly differentially expressed genes in the control and PM-N groups. Among the 20 genes significantly upregulated in the PM-N group, at least half were involved in cell migration, proliferation, and neural differentiation. The Vcan and Fstl5 genes regulate cell adhesion, proliferation, differentiation, migration, and angiogenesis. Genes such as Grin3a, Dscam, Dll1/3, and Pllppr1 play crucial roles in nervous system development and neuronal plasticity. Shisa7, Gabbr2, and Sox8 are associated with receptors of GABAergic neurons. Venn diagrams and volcano plots were generated to illustrate the total number of differentially expressed genes (Figure 5B-C). Kyoto Encyclopedia of Genes and Genomes (KEGG) enrichment analysis revealed the top 20 pathways enriched in the control and PM-N groups (Figure 5D). Pathways such as the calcium signaling pathway, GMP-PKG signaling pathway, cholinergic synapse, cAMP signaling pathway, synaptic vesicle cycle, axon guidance, GABAergic synapse, and glutamatergic synapse were related to NSC differentiation and regeneration. Furthermore, gene ontology (GO) analysis revealed that the upregulated genes were associated with the regulation of synaptic plasticity, axonogenesis, positive regulation of synaptic transmission, neuron differentiation, synapse assembly, synapse organization, transsynaptic signaling regulation, and chemical synaptic transmission modulation. Conversely, downregulated genes were linked to positive regulation of cell migration and motility (Figure 5E-F). Notably, the downregulation of genes involved in extracellular matrix organization, vasculature development regulation, peptidase activity regulation, endopeptidase activity regulation, and the wound response may be attributed to the effects of NM. Considering the results from heatmaps, KEGG enrichment analysis, and GO analysis, the cGMP-PKG and cAMP signaling pathways are likely mechanisms through which NABDM promotes NSC neural differentiation. Western blot and statistical analyses were conducted to detect key proteins in these pathways. As shown in Figure 5G-H, the protein expression levels of p-ERK/ERK in the PM-N group were greater than those in the control group. Therefore, we propose that NABDM induces the neural differentiation of NSCs by activating the cGMP-PKG and cAMP signaling pathways. The cGMP-PKG pathway is associated with calcium channels, neurotransmitter release, and GABAergic neuron receptors, whereas the cAMP signaling pathway is related to the regulation of membrane protein activity and synaptic transmission conditions. These findings are consistent with the aforementioned results.

### NABDM inhibits inflammation and promotes NSC differentiation and maturity in SCI mice

**Figure 6:**
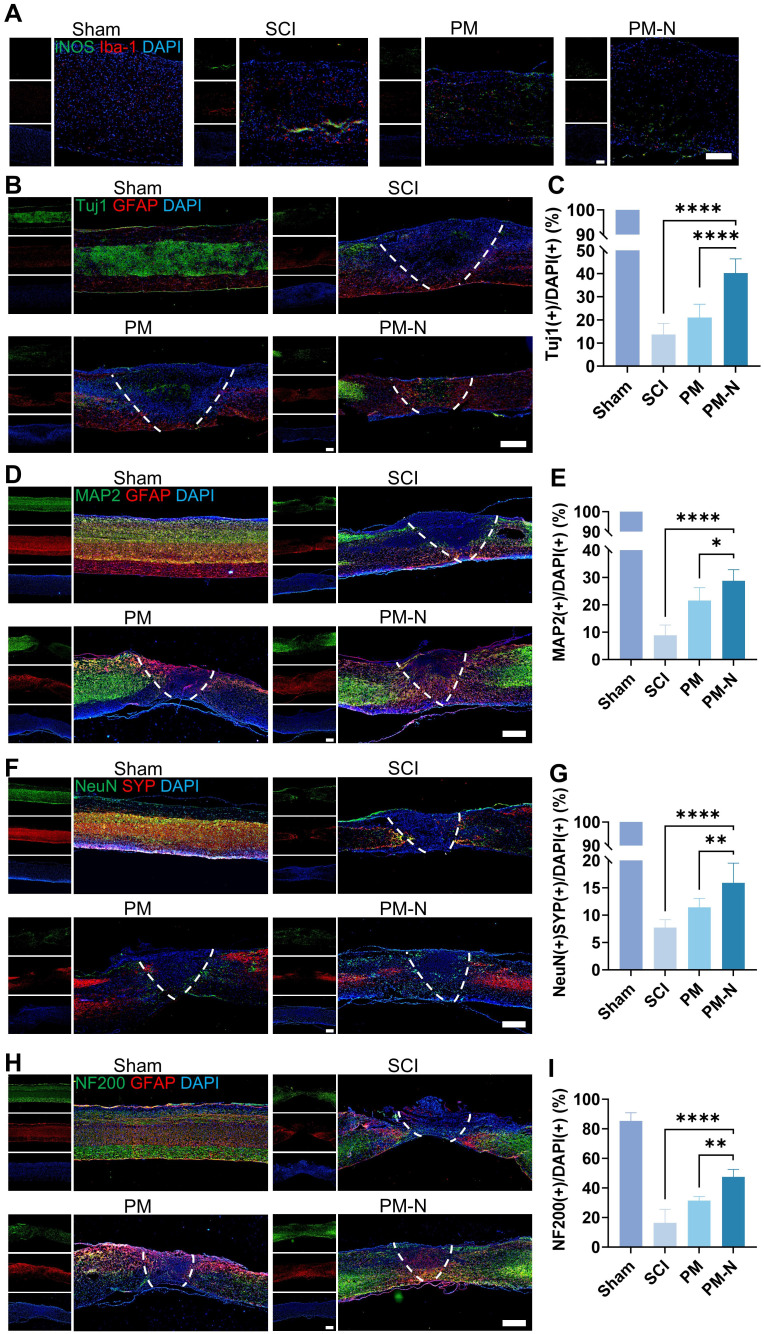
NABDM inhibit local inflammation and promote NSC differentiation after SCI. A) Immunofluorescent staining of iNOS/Iba-1 in different groups at 1 day postinjury. Scar bar, 250 μm. B and C) Immunofluorescent staining and analysis of Tuj1/GFAP in different groups at 7 days postinjury. Scar bar, 500 μm. n=6, ****p < 0.0001. D, E) Immunofluorescent staining and analysis of MAP2/GFAP in different groups at 28 days postinjury. Scar bar, 500 μm. n=6, *p < 0.05, ****p < 0.0001. F, G) Immunofluorescent staining and analysis of NeuN/SYP in different groups at 28 days postinjury. Scar bar, 500 μm. n=6, **p < 0.01, ****p < 0.0001. H, I) Immunofluorescent staining and analysis of NF200/GFAP in different groups at 28 days postinjury. Scar bar, 500 μm. n=6, **p < 0.01, ****p < 0.0001.

An SCI contusion model was used to investigate the role of NABDM in the *in vivo* environment of NSCs (Figure S8). Inflammation at the injury site was assessed by immunofluorescence staining for iNOS and Iba-1 one day postinjury. The results revealed decreases in iNOS- and Iba-1-positive cells in the PM-N group compared with those in the SCI group (Figure 6A). NSC differentiation was evaluated through immunofluorescence staining for Tuj1 and GFAP at 1 and 7 days postinjury. At 1 day postinjury, there was nearly no difference between the groups. However, at 7 days postinjury, the PM-N group presented a greater number of immature neurons marked by Tuj1 (Figures 6B-C and S9). Neuron differentiation was further assessed using MAP2, a marker for mature neurons, 28 days postinjury. The immunofluorescence results revealed a greater ratio of MAP2-positive cells in the PM-N group than in the other groups (Figure 6D-E). The formation of local neuronal circuits, which is indicative of neurobehavioural function recovery, was evaluated using synaptophysin (SYP), a presynaptic marker, and NeuN, another mature neuron marker, at 28 days postinjury 28. The PM-N group presented greater numbers of both SYP-positive and NeuN-positive cells (Figure 6F-G), indicating an increased presence of synaptic junctions. To further observe neuronal circuit formation and axon development, NF200, a marker for heavy chain neurofilaments, was stained at 28 days postinjury. The results revealed a greater number of nerve fibers in the PM-N group (Figure 6H-I). These findings demonstrate that, compared with the other groups, the NABDM group presented reduced inflammation, enhanced NSC differentiation, and an increased number of mature neurons.

### NABDM enhance function recovery of mice after SCI

**Figure 7:**
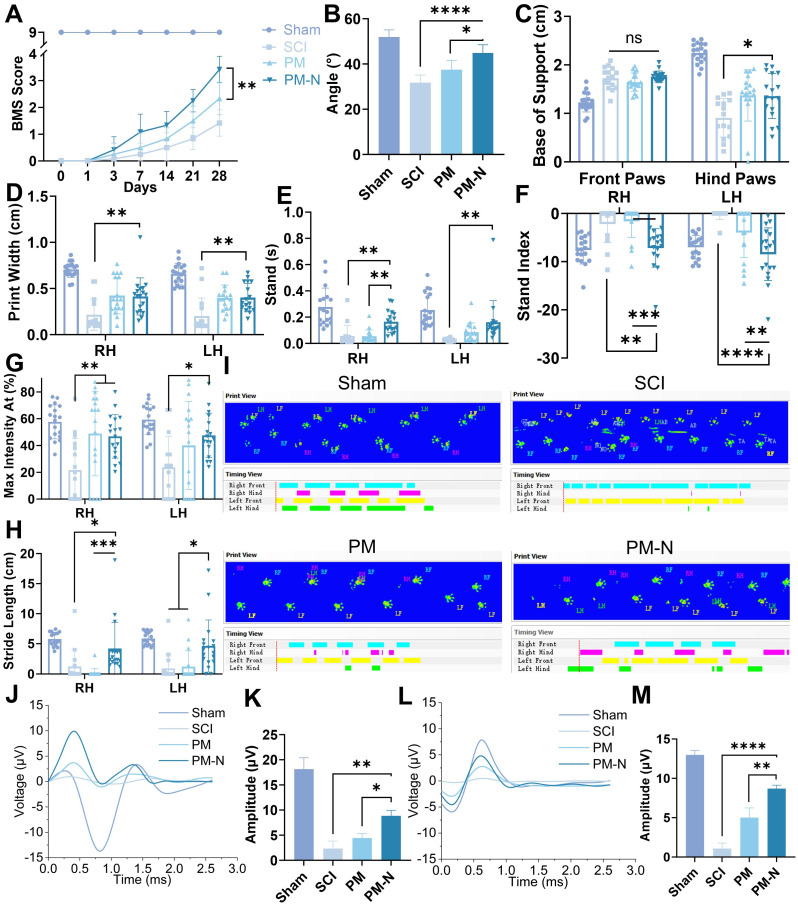
NABDM promote motor function recovery after SCI. A) The BMS score at 28 days postinjury. n=6, **p < 0.01. B) The inclined plate test at 28 days postinjury. n=6, *p < 0.05, ****p < 0.000. C-H) The analysis of CatWalk test at 28 days postinjury. n=18, *p < 0.05, **p < 0.01, ***p < 0.001, ****p < 0.0001, ns, not significant. I) The print of CatWalk test at 28 days postinjury. J and K) The result and analysis of SEP at 28 days postinjury, *p < 0.05, **p < 0.01. L and M) The result and analysis of MEP at 28 days postinjury. n=3, **p < 0.01, ****p < 0.0001.

The locomotor behaviour of C57BL/6 mice was assessed using the inclined plate test and the Basso Mouse Scale (BMS) score, both of which are commonly used to evaluate motor and systemic recovery 29. The mice in the PM-N group exhibited superior hind limb motor performance, maintaining a larger angle on the inclined plate, reaching up to 44° (Figure 7A-B, and S10). Specifically, more mice in the PM-N group scored 3 or 4 on the BMS at 28 days postinjury, demonstrating dorsal stepping and temporary body support, than did the mice in the SCI and PM groups, which mainly exhibited ankle movement. Gait improvement post-SCI was analysed using the CatWalk system at 28 days postinjury. The mice in the PM-N group displayed more regular gait patterns and improved print width, base of support, stand, stride length, stand index, and maximum contact area metrics (Figure 7C-I and S11). These results indicated more stable standing, greater foot support area, and more regular gait during walking, whereas the gait patterns in the SCI and PM groups were more disorganized than those in the PM-N group. The functional improvement was further corroborated by electrophysiological measurements, including somatosensory evoked potential (SEP) and motor evoked potential (MEP), which reflect the levels of sensory and motor electrical activity in the spinal cord. At 28 days postsurgery, both the SEP and MEP amplitudes were more pronounced in the PM-N group, with the SEP amplitude in the PM-N group reaching 8.9 µV and the MEP amplitude reaching 8.7 µV. Although there was no significant difference in the latency period between the groups, except for that of the SCI group, which was slightly longer, this difference may be attributed to the direct placement of electrodes on the sciatic nerve and brain. (Figure 7J-M and S12) These results demonstrated that, compared with those in the other groups, the levels of locomotor function and sensory recovery of the animals treated with NABDM were superior.

### NABDM enhances sensory and autonomic function recovery in mice after SCI

**Figure 8:**
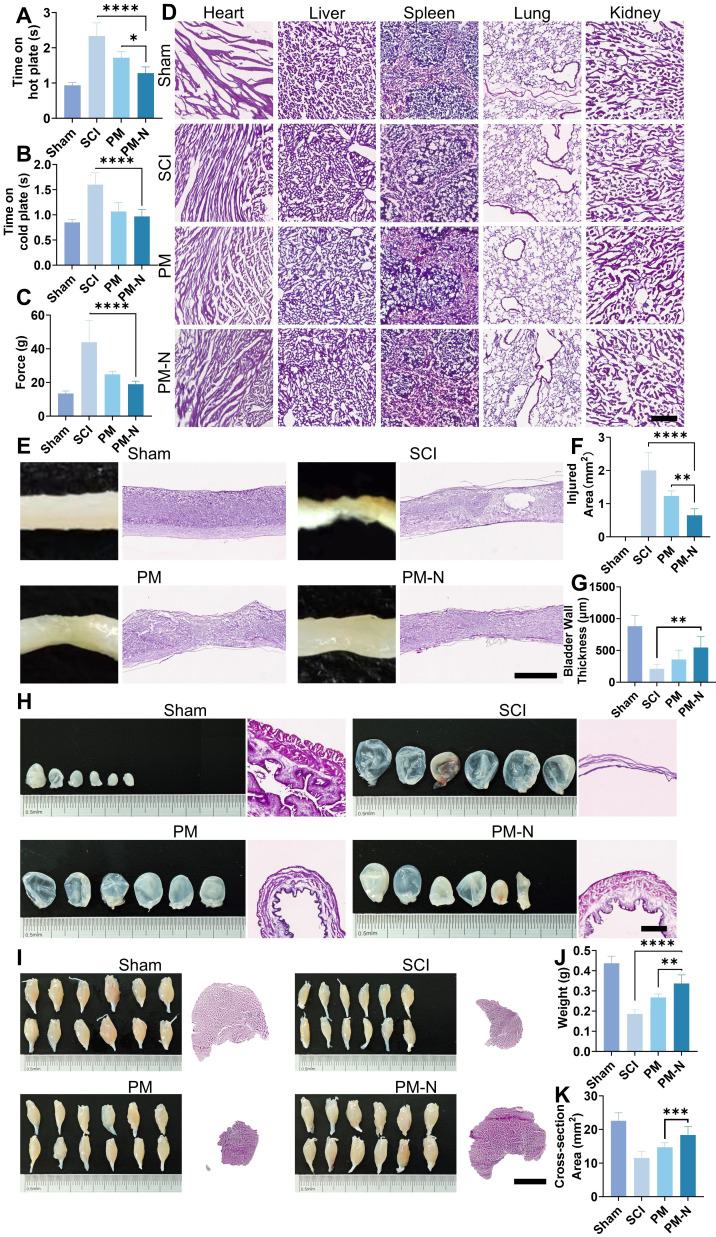
NABDM promote sensory function and autonomic function recovery after SCI. A, B and C) The hot-cold plate test and Von Frey test for sensory function at 28 days postinjury. n=6, *p < 0.05, ****p < 0.0001, ns, not significant. D) H&E stains for heart, liver, spleen, lung and kidney in different groups at 28 days postinjury. Scar bar, 250 μm. E and F) The general photograph, H&E staining and analysis of injury area at 28 days postinjury. Scar bar, 1 mm. n=6, **p < 0.01, ****p < 0.000. G, H) Recovery of bladder in different groups at 28 days postinjury. Scar bar, 500 μm. n=6, **p < 0.01. I, J and K) Recovery of muscle in different groups at 28 days post-injury. Scar bar, 2.5 mm. n=6, ***p < 0.001.

To further confirm the efficacy of NABDM in treating SCI, a series of tests, including sensory assessments and H&E staining, were conducted on the spinal cord, bladder, and muscle. Sensory function was evaluated using hot and cold plate tests and the Von Frey test, which indicated that the PM-N group presented better sensory function (Figure 8A-C). Mice in the PM-N group were more sensitive to low temperatures, reacting within 1 s on the hot plate, compared to mice in the control group, which reacted within an average of 1.3 s. The safety of NABDM was also assessed *in vivo*. In a preliminary experiment, a blank membrane was placed at the SCI site in rats, followed by MRI examination. The results revealed no significant increase in the local response compared with that in the SCI group, confirming the good biocompatibility of the fibrous membrane (Figure S13). For the formal experiment, the mice were evaluated during the refinement treatment. H&E staining of the heart, liver, spleen, lung, and kidney revealed no distinct differences between the groups, indicating favourable biocompatibility of NABDM (Figure 8D). General photographs and H&E staining of the spinal cord tissue revealed that the PM-N group had a smaller injury area than the other groups did (Figure 8E-F). H&E staining has also been used to evaluate autonomic nervous system function, with a focus on amyotrophy and bladder dysfunction, which are major concerns for patients with SCI 30. The condition of the gastrocnemius muscle and bladder was assessed at 28 days postinjury. The photographs and tissue sections in the PM-N group generally revealed stronger muscle and smaller bladders. There were significant differences in muscle cross-sectional area, muscle weight, and bladder thickness between the PM-N group and the other groups, with measurements reaching 18.3 mm², 0.34 g, and 548.3 µm, respectively (Figure 8G-K). In conclusion, NABDM demonstrated a superior ability to promote recovery from SCI and exhibited good biocompatibility.

## Methods

### Preparation of NABDM

PLGA (50:50, Mw = 880,000-1,170,000) and PEG (Mw = 44.05) were used as shell matrices, and the mass percentages of PEG in the shell matrices of each group were 0, 5, 10, and 20%. The shell matrix and hexafluoroisopropanol were mixed and dissolved at a mass/volume ratio of 1:5. NM was added to the drug-loaded group at a mass ratio of NM to a shell matrix of 1:10, and ultrasonic dispersion was performed for 1 min. NM was not added to the nondrug-loaded group to obtain shell liquid. PLA (Mw = 100,000-130,000) and hexafluoroisopropanol were mixed and dissolved at a mass/volume ratio of 1:8. NT3 was added to the drug-loaded group at a ratio of 1 μg of NT3 per 0.1 g of PLA, and ultrasonic dispersion was performed for 1 min. NT3 was not added to the nondrug-loaded group to obtain the core liquid. A coaxial electrospinning nozzle was used to connect two independent syringes of core liquid and shell liquid. The inner tube diameter of the spinning nozzle was 0.40 mm, the flow rate was 0.5 mL/h, the outer tube diameter was 0.72 mm, the flow rate was 2 mL/h, the ambient temperature was 25 °C, the ambient humidity was 65%, the distance from the spinning nozzle to the receiver was 18 mm, and the spinning time was 3 h. The samples were collected on aluminium foil, freeze-dried for 24 h, and stored at 4 °C when used and -20 °C when stored 31.

### Characterization of NABDM

The methods used for the SEM, TEM, EDS, FTIR and hydrophilicity tests were as described above 32-36.

### Extraction and culture of NSCs, BV2 cell culture and group *in vitro*

C57/BL6 mouse (Vital River) embryos (E14) were used to extract NSCs. Briefly, the cerebral cortex of each embryo was isolated, and the cortical tissue was isolated without vascular tunica and enzymatically digested with DNase I (Sigma) and papain (Worthington Biochemical) to extract the NSCs. The cell suspension was then passed through a 40-μm cell strainer to eliminate any undigested tissue debris. After centrifugation, the cells were resuspended in culture medium consisting of 1% penicillin/streptomycin, 1% GlutaMAX™-1, 2% B27 supplement without vitamin A, 96% neurobasal medium, 20 ng/mL bFGF and EGF (all from Gibco), and the suspensions were cultured in cell culture flasks. NSCs were subjected to two passages to ensure the purity of the NSCs for subsequent cell experiments. BV2 cells were cultured in DMEM-HG (Gibco) supplemented with 10% FBS and 1% penicillin/streptomycin. The cells were allocated into three groups: (1) the control group, (2) the PLGA(PEG-20%)/PLLA membrane group (PM group), and (3) the PLGA(PEG-20%)/PLLA + NM + NT3 membrane (NABDM) group (PM-N group).

### Cytocompatibility evaluation

A CCK-8 assay was conducted to measure the viability and proliferation of the different groups. Briefly, 2 × 10^4^ NSCs were seeded onto 0.01% poly-D-lysine-precoated 48-well culture plates and cultured for 1, 2, or 3 days. At the specified time points, the medium in the 48-well plates was replaced with neurobasal medium containing 10% CCK-8 solution and incubated at 37 °C in a 5% CO_2_ atmosphere for 2 h. The medium was then transferred to a 96-well plate and analysed using a microplate reader at 450 nm. A live/dead staining assay was performed to assess cytocompatibility. Briefly, 4 × 10^4^ NSCs were seeded onto 48-well culture plates and cultured for 1 day. The medium was then replaced with PBS containing 4 μM propidium iodide (PI) and 2 μM calcein AM. After a 10 min incubation at 37 °C, the samples were observed using laser confocal microscopy.

### RT-qPCR

NSCs (2 × 10^6^) were seeded onto 0.01% poly-D-lysine-precoated 6-well culture plates subjected to different treatment. Following the culture period, the cells were resuspended in culture medium consisting of 1% FBS, 1% penicillin/streptomycin, 1% GlutaMAX™-1, 2% B27 supplement, and 96% neurobasal medium (Gibco). There were no special requirements to BV2 cells. The cells were then lysed using TRIzol reagent (Invitrogen), and total RNA was extracted according to the manufacturer's instructions. A PrimeScript™ RT reagent kit (Perfect Real Time) (Takara) was used to reverse transcribe the total RNA into cDNA. RT‒qPCR was subsequently performed to measure the transcription levels of neurogenesis-related genes. The reaction mixture included TB Green® Premix Ex Taq™ II (Tli RNaseH Plus), specific primers for the target genes, and cDNA.

### Western blot analysis

Proteins from cells in different groups were lysed in a mixture containing RIPA lysis buffer, a protease inhibitor, and a phosphatase inhibitor (Solarbio). After centrifugation, the proteins were denatured with 5× sodium dodecyl sulfate loading buffer and separated by 10% SDS‒PAGE (Solarbio). The proteins were then transferred to a 0.22 μm PVDF membrane (Invitrogen) and blocked with 5% BSA (Beyotime). The membrane was incubated with primary antibodies overnight at 4 °C, followed by incubation with secondary antibodies for 1 h at room temperature. A ChemiDoc MP imaging system (Bio-Rad) and chemiluminescence (Invitrogen) were used for exposure imaging.

### Immunofluorescence staining of cells

NSCs (2 × 10^5^) were seeded onto 14 mm diameter tissue culture plates precoated with 0.01% poly-D-lysine and placed in 24-well culture plates with various treatment. There were no special requirements to BV2 cells. After fixation with 4% paraformaldehyde, the cells were permeabilized with 0.1% Triton X-100 and blocked with a 10% BSA solution. The cells were subsequently incubated with primary antibodies overnight at 4 °C, followed by incubation with secondary antibodies for 1 h at room temperature. Finally, the nuclei were stained with DAPI, and the samples were observed using laser confocal microscopy and fluorescence microscopy.

### RNA sequencing and data analysis

TRIzol reagent was used to extract total RNA from NSCs subjected to different treatments following the manufacturer's instructions. Novogene (Beijing, China) performed the RNA sequencing of the obtained RNA samples. An NEBNext Ultra™ Directional RNA Library Prep Kit for Illumina was utilized to generate sequencing libraries. The index of the reference genome was built using HISAT2 (v2.0.5), and paired-end clean reads were aligned to the reference genome using HISAT2 (v2.0.5). Following the alignment, featureCounts (v1.5.0-p3) was employed to analyse differential gene expression, utilizing the BAM files obtained from each individual alignment.

### SCI surgery

Animal experiments were conducted with the approval of the Laboratory Animal Ethics and Welfare Committee of Shandong University Cheeloo College of Medicine (Approval number: 23096; Animal use permit number: SYXK: 20230003). Four experimental groups were established: (1) the Sham group, (2) the SCI group, (3) the PLGA(PEG-20%)/PLLA membrane group (PM group), and (4) the PLGA(PEG-20%)/PLLA + NM + NT3 membrane (NABDM) group (PM-N group). Female C57BL/6 mice aged 6-8 weeks were used to establish a spinal cord contusion model. All the mice were anaesthetized with isoflurane. Following a midline incision and blunt dissection of the tissue above the T9 vertebral laminae, a T9 laminectomy was performed. The spinal cord was then subjected to a medial stroke using Feng's Standard Strike device. The mice were then treated with the respective membranes. Postsurgery, the mice received manual bladder expression twice a day and a daily injection of cefuroxime for seven days.

### Basso Mouse Scale (BMS) score

BMS scores were used to assess the motor behaviour of the hindlimbs. Locomotion of the hindlimbs was evaluated on a scale ranging from 0 (no ankle movement) to 9 (complete functional recovery). All the mice were assessed by blinded observers on days 1, 3, 7, 14, 21, and 28 postsurgery if they were evaluated with a score of 0 immediately after surgery.

### Catwalk analysis

A Catwalk XT® automated gait analysis system (Noldus Information Technology, The Netherlands) was used for gait assessment. All the mice were assessed at 28 days based on an average of three steps in a row.

### Electrophysiology

At 28 days postsurgery, the mice were anaesthetized with isoflurane. SEP and MEP were recorded using electrophysiological devices. Under anaesthesia, the sciatic nerve and sensorimotor cortex of each mouse were exposed. For SEP recordings, a stimulator was inserted into the sciatic nerve, whereas for MEP recordings, a stimulator was placed on the sensorimotor cortex. A constant current intensity of 10 mA for SEP and 15 mV for MEP was applied with a 0.05 ms pulse width.

### Mouse sensory evaluation

The hot and cold plate tests were conducted 28 days postsurgery. The hot and cold plates (BIO-CHP, Bioseb) were preheated to 55 °C and precooled to 1 °C. Once the target temperature was reached, the mice were placed on the plates, and timing began. The timing started when the mouse was placed on the plate and ended when the leg twitched or jerked. A period of rest was allowed between the hot and cold tests to prevent overreaction. The Von Frey test (BIO-EVF4, Bioseb) was also performed 28 days postsurgery. A plastic needle was connected to the electronic pain meter sensor, and the fixed hind leg of each mouse was exposed. The middle of the foot was pressed, and the force was gradually increased until the mouse exhibited a reaction, such as fingertip movement, foot flexion, or foot retraction. Another experimenter recorded the value displayed by the instrument, and each mouse was tested three times, with the lowest value recorded.

### Tissue staining

After being deeply anaesthetized, the mice were perfused with precooled PBS followed by 4% paraformaldehyde. The spinal cords were then fixed, dehydrated, embedded in paraffin, and sectioned. The sections were washed three times with TBST and then blocked with a solution containing 5% normal goat serum, 0.1% BSA, and 0.25% Triton X-100 in TBS. The samples were subsequently incubated with primary antibodies diluted in blocking buffer overnight at 4 °C. After being washed with TBST, the sections were incubated with the appropriate secondary antibodies for 2 h at room temperature. Following additional washes, the sections were cover-slipped and imaged using laser confocal microscopy and fluorescence microscopy. For H&E staining, the sections were stained with haematoxylin and eosin after sectioning and then photographed using a microscope.

### Statistical analysis

The relative protein expression levels and fluorescence intensities of the images were analysed with ImageJ software. All values are presented as the means ± standard deviations (SDs). Statistical analyses were conducted using GraphPad Prism 8.0 software. Statistical significance was determined using Student's t tests or one-way ANOVA with the Bonferroni comparison test. Significance levels of differences: *p < 0.05, **p < 0.01, ***p < 0.001, ****p < 0.0001, ns, not significant.

## Discussion

In this study, NABDM was synthesized using coaxial electrospinning, and core-shell structured nano-fiber/nets were formed. Compared with a single structure, the core-shell structure can ensure that the two drugs can be released during the corresponding time period of SCI, avoiding the side effects caused by drug overdose, whereas the nano-fiber/net structure can improve the mechanical strength changes caused by drug loading. The material consists of an outer layer of PLGA embedded with NM, which inhibits neuroinflammation, and an inner layer of PLLA embedded with NT3, which promotes the differentiation of NSCs by activating the cGMP-PKG and cAMP signaling pathways. Compared with other materials, PLGA and PLLA are FDA-approved materials with excellent biosafety and can be adapted for a variety of technical processes. PLGA is used as the outer layer structure with fast degradation speed and good stability, whereas PLLA is used as the inner layer structure with slow degradation speed and can provide support. Compared with other forms of PLGA and PLLA, electrospinning membranes can be more stable at sites of injury, provide some support while slowing the release of drugs, and avoid side effects such as liver damage 21, 22. Therefore, both PLGA and PLLA facilitate the gradual release of drugs. Initially, NM is released to create an optimal environment for differentiation, serving an "external" function and setting the stage for NSC differentiation. The "oral" delivery of NT3 subsequently directly enhances NSC differentiation. This sequential, four-dimensional approach fully promotes NSC differentiation, offering a novel differentiation-promoting strategy. The NABDM demonstrates excellent biocompatibility and mechanical strength comparable to those of the spinal cord dura mater. When implanted into an SCI contusion model mouse, an NABDM reduces local neuroinflammation, accelerates endogenous NSC differentiation, and increases the number of mature neurons, thereby improving motor, sensory, and autonomic nerve functions; reducing the SCI area; and promoting recovery. The fabrication of an NABDM represents a new, rapid, and effective treatment for SCI. Its preparation is straightforward, and its application is simple and rapid, making it suitable for clinical use as a patch. The NABDM is particularly valuable for the prehospital emergency treatment of patients with acute SCI and holds promising prospects for clinical translation and application.

In previous studies, researchers have used transcription factors and mRNAs to address the persistent challenge of NSC differentiation 37, 38. These interventions aimed to accelerate NSC differentiation and direct their fate towards becoming neurons rather than astrocytes, thereby increasing the production of neuronal cells. However, targeting these specific interventions often results in broader systemic effects. Additionally, some researchers have explored physical signals, such as electrical, mechanical, and optical signals, that influence NSC differentiation 39-43. Despite these efforts, the *in vivo* application of these methods faces numerous obstacles, including uncertain outcomes, an uncontrolled intervention range, and biocompatibility issues. Moreover, some studies have failed to consider the timing and cellular environment of NSC differentiation *in vivo*, limiting their effectiveness. In this study, the neurotrophic factor NT3, which primarily targets NSCs while minimizing their impact on other cells, was utilized to promote NSC differentiation. Initially, the NM in the outer layer of the core-shell structure was released to reduce neuroinflammation caused by microglia, creating a favourable early-stage environment for NSC differentiation. NT3 was subsequently released to directly promote NSC differentiation at the appropriate time. The development of drug delivery systems has been a key research focus, with significant progress made in hydrogels and nanoparticles 44-47. However, challenges remain 48. Although hydrogels have high water content, good tissue toughness, and degradability, they often have uncontrolled drug release rates, leading to excessive local drug concentrations 49, 50. Nanoparticles made from metals or polymers can prolong drug action but face challenges such as being fixed in specific areas, being lost through cellular or tissue metabolism, and causing toxicity due to liver aggregation 51. This study employs coaxial electrospinning technology to create a nano-fiber/net membrane delivery system. The system can locally fix and release drugs in a sustained manner through the core-shell structure of the nanofibers, addressing various aspects of SCI and enhancing therapeutic effects. However, researchers typically focus on biological properties, with the result that the mechanical properties of materials are often overlooked 52, 53. The dura mater surrounding the spinal cord provides essential mechanical support, and this study emphasized this aspect. Previous studies have indicated that the mechanical strength of human corpse dura mater exceeds 1 MPa, with that of mouse dura mater reaching 1.9 MPa and that of bovine dura mater reaching approximately 2 MPa 54, 55. The artificial dura mater developed in this study had a mechanical strength of approximately 2.2 MPa, meeting the requirements of various organisms. The addition of drugs and the use of coaxial electrospinning resulted in a nano-fiber/net structure, which was initially used in air filtration and sensor research 25. This structure is an emerging direction in electrospinning, with limited biomedical research focused primarily on wound healing and drug release 26, 27. In this study, this structure was applied for SCI repair, improving membrane tensile strain and facilitating cell adhesion and drug release, thus expanding the scope of application.

Despite the successful manufacture of artificial dura mater, challenges remain. This study primarily explored the mechanisms promoting NSC differentiation via the cGMP-PKG and cAMP pathways but did not investigate downstream pathways, such as the Notch pathway, which is related to neural differentiation, or other pathways involving ERK 56. In the animal experiments, the duration of mouse observation after SCI was relatively short. Although researchers often observe 4-6 weeks post-SCI, long-term studies extending to 3-6 months are necessary to assess chronic SCI recovery, including urination function and mood.

Regarding the limitations and shortcomings of this study, the NABDM lacks the ability to treat both sides and the ventral side of the injured spinal cord and can perform only 2D-level treatment due to limitations in surgical techniques and equipment. In addition, the mechanism of the NABDM has not been explored in depth; future research should delve deeper into the mechanisms, explore whether the cGMP-PKG and cAMP pathways operate through calcium channels, the role of neurotransmitter conduction position channel opening, and methods to increase the expression levels of neuron-related proteins, such as Tuj1 and MAP2 57, 58. Epigenetic modifications of proteins may play crucial roles and warrant further study. Long-term observation indicators post-SCI need to be clarified, with urodynamic and muscle strength tests serving as important evaluation criteria, as standardized scoring rules for small animals are still lacking.

Artificial bionic dura mater holds promising application prospects, effectively repairing contused spinal cords but requiring further optimization. Clinically, it can be used as an emergency treatment or fixed to the site of injury by microsuturing for long-term sustained release, necessitating increased operator skills. This study discussed its application in a mouse SCI model. For clinical translation, the thickness and mechanical properties of the membrane may need adjustment to match the mechanical strength of the human dura mater, which is greater than 20 MPa 54. Future studies may explore three- or even four-layer core-shell nanofibers for long-term four-dimensional treatment. After the acute phase of SCI, inner-layer drugs can be released to aid in chronic phase recovery, potentially through the use of drugs that promote axon regeneration or stimulate motor neuron differentiation. Multilayered membrane structures or nanozymes tailored for different SCI stages are also viable 59, 60. Additionally, further research into the mechanisms promoting NSC differentiation is essential. Although the roles of the NOTCH signaling pathway and proteins such as DLL3, DLL4, and HES1/5 in NSC differentiation have been established, their regulation of proteins such as Tuj1 and MAP2 remains unclear 61, 62. Direct regulation of neuron-related proteins is a critical next research direction.

## Conclusion

In this study, we utilized coaxial electrospinning technology to successfully prepare NABDM with mechanical properties comparable to those of natural dura mater, incorporating NM and NT3. Cellular experiments demonstrated that the NABDM exhibited excellent biocompatibility, modified the polarization state of BV2 cells, and promoted the differentiation of NSCs. The promoting effect on differentiation was likely mediated through the activation of the cGMP-PKG and cAMP signaling pathways. Upon implantation in a mouse contusion model of SCI, the NABDM facilitated SCI recovery by reducing local inflammation, accelerating the differentiation of endogenous NSCs, and increasing the number of mature neurons. This resulted in improvements in motor, sensory, and autonomic nerve functions, as well as a reduction in the SCI area, thereby demonstrating the biocompatibility of the fiber membrane. The results of this study provide a novel, rapid, and effective method for treating SCI.

## Supplementary Material

Supplementary figures.

## Figures and Tables

**Figure 1:**
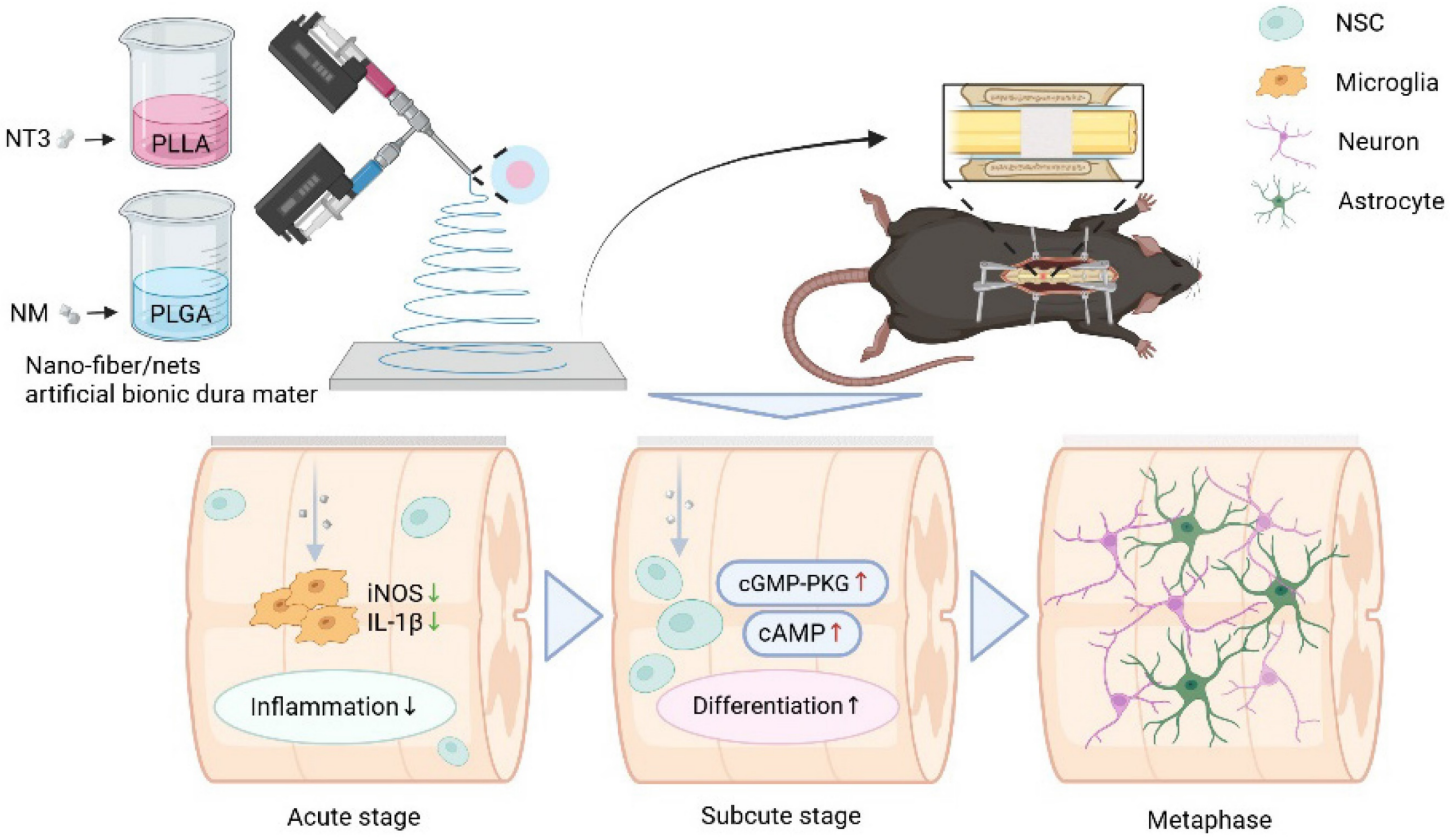
Preparation of NABDM and its role in SCI treatment. NABDM reduces inflammation by modulating microglia polarization and accelerate NSC differentiation through cGMP-PKG and cAMP signaling pathways, thereby facilitating SCI repair.

## Abbreviations

BMS: basso mouse scale
cAMP: cyclic adenosine monophosphate
cGMP-PKG: cyclic guanosine monophosphate- protein kinase G
ERK: extracellular regulated protein kinases
FDA: food and drug administration
FTIR: fourier transform infrared spectroscopy
GFAP: glial fibrillary acidic protein
GO: gene ontology
H&E: hematoxylin-eosin
IL-1β: interleukin-1β
iNOS: inducible nitric oxide synthase
KEGG: kyoto encyclopedia of genes and genomes
LPS: lipopolysaccharide
MAP2: microtubule-associated protein 2
MEP: motor evoked potential
mRNA: messenger ribonucleic acid
NABDM: nano-fiber/net artificial bionic dura mater
NeuN: neuronal nuclei
NF200: neurofilament heavy chain
NM: nafamostat mesylate
NSC: neural stem cell
NT3: neurotrophins-3
PEG: polyethylene glycol
PLGA: poly(lactic-co-glycolic acid)
PLLA: poly(l-lactic acid)
RT-qPCR: real-time quantitative polymerase chain reaction
SCI: spinal cord injury
SEM: scanning electron microscope
SEP: somatosensory evoked potential
SYP: Synaptophysin
TEM: Transmission electron telescope
Tuj1: beta-tubulin III
