# Supplementary material for: Nano-fiber/net artificial bionic dura mater promotes neural stem cell differentiating by time sequence external-oral administration to repair spinal cord injury
Source: Theranostics. 2025 Jan 27;15(6):2579–96. doi: 10.7150/thno.102584 (PMC11840742; doi:10.7150/thno.102584)
Supplement: Supplementary file 1 — Supplementary figures. [file thnov15p2579s1.pdf]

Supporting information

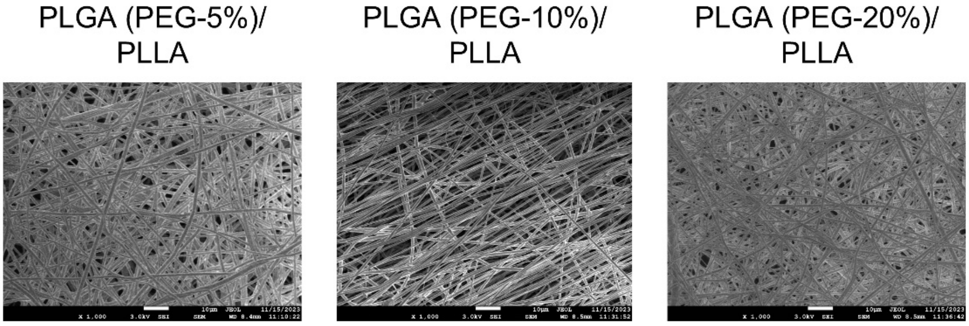

Figure S1. The SEM of membrane modified by PEG.

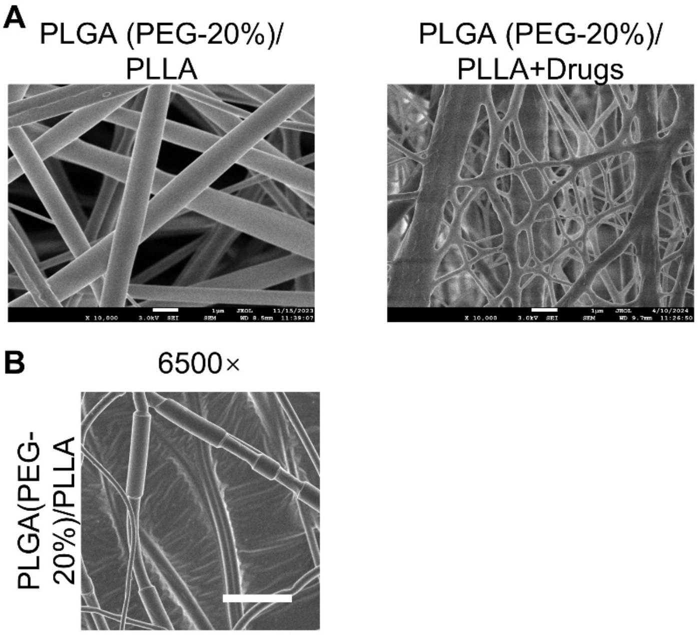

Figure S2. A) The SEM of membrane with or without drugs. B) The SEM of PLGA(PEG-20%)/PLLA core-shell structure membrane. The scar bar is 4 μm.

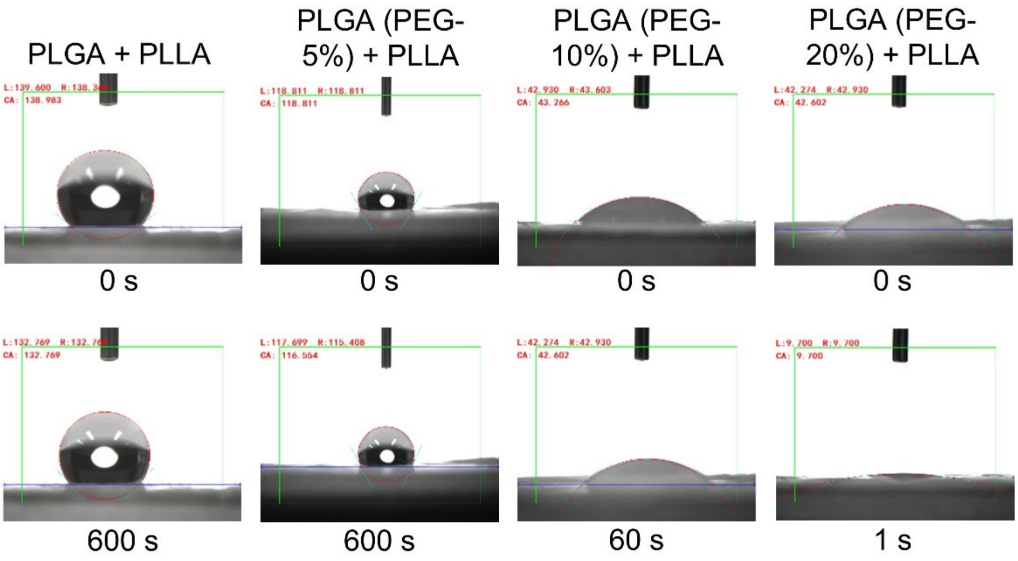

Figure S3. The hydrophilia of membrane with different content PEG.

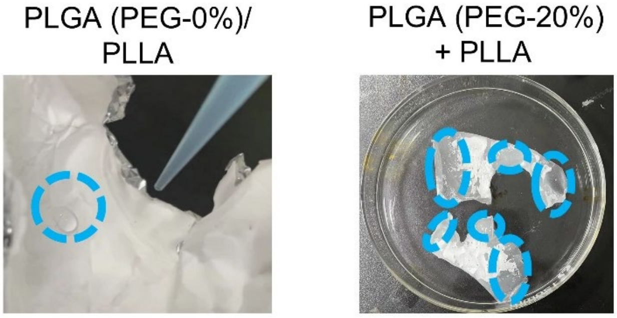

Figure S4. The general photograph of hydrophilia after modified with PEG.

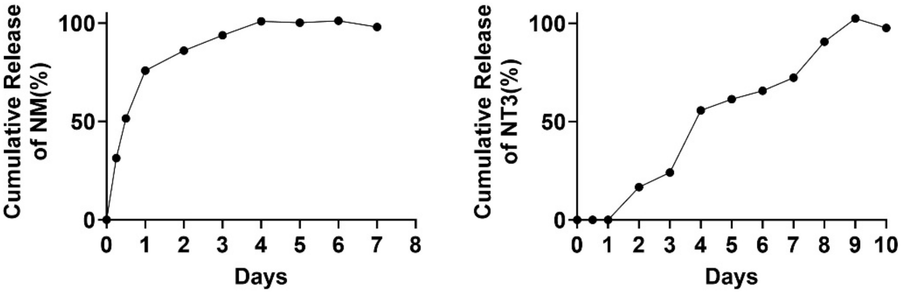

Figure S5. Sustained drug release of NM and NM3 in NABDM.

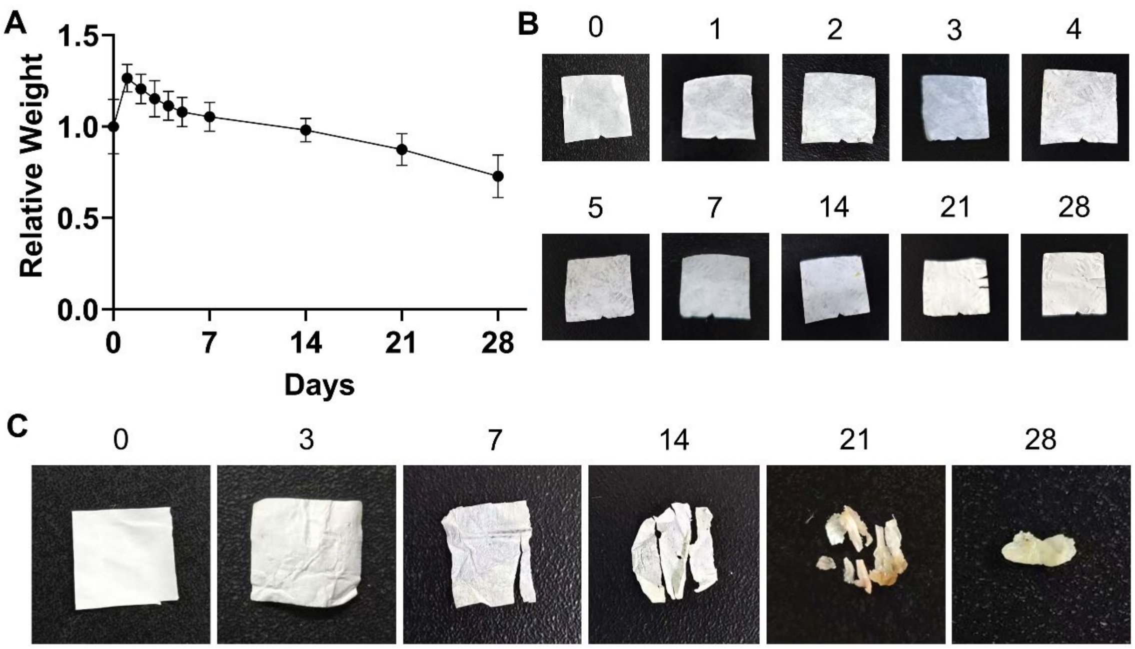

Figure S6. Degradation of NABDM with a size of 1.5 cm×1.5 cm in vivo and in vitro. A, B) Weight and appearance of NABDM in vitro hydrolysis. C) Appearance of NABDM degradation in subcutaneous implantation. n=3

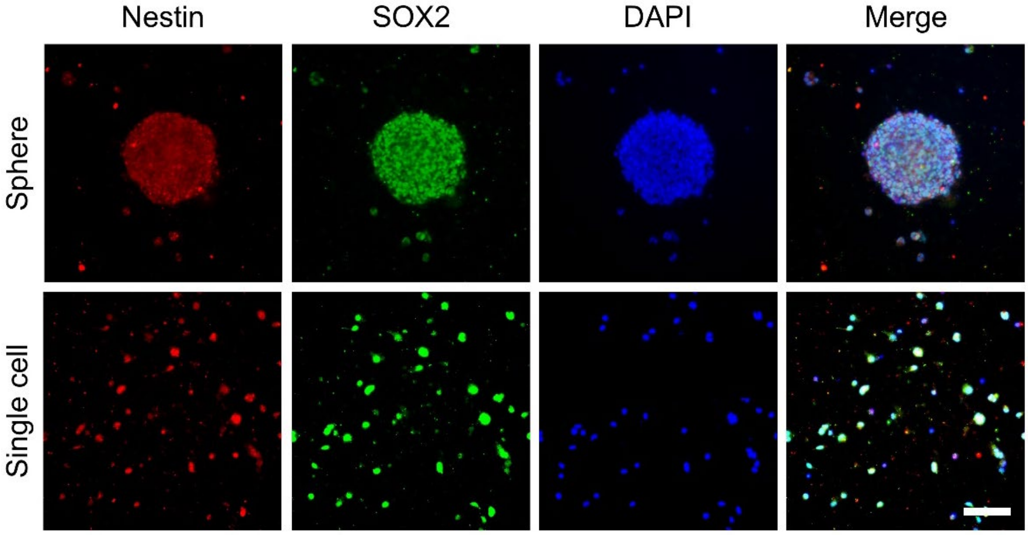

Figure S7. Immunofluorescence staining identification of NSCs. Scar bar, 100 μm.

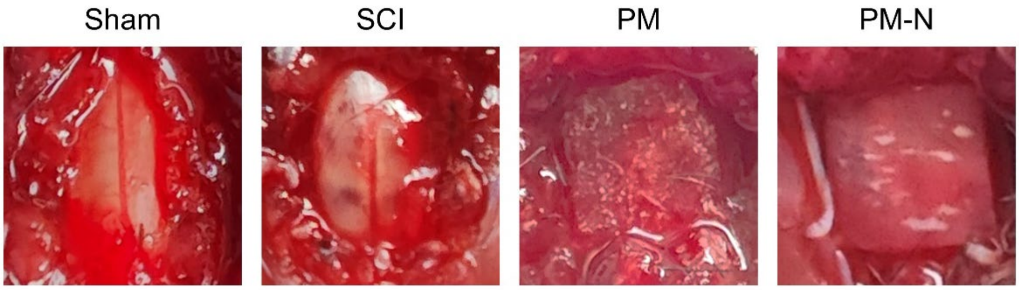

Figure S8. The general photograph of SCI model with different treatment.

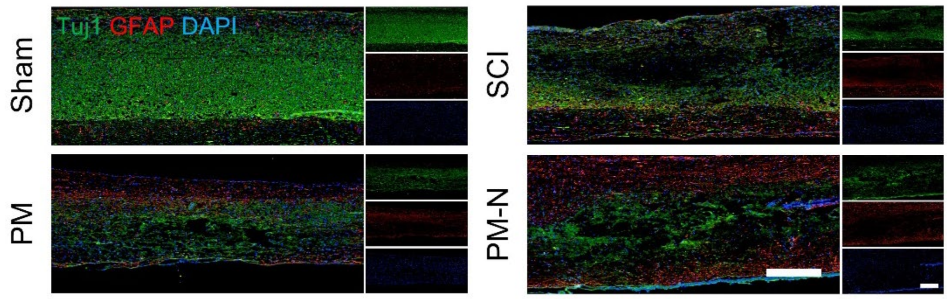

Figure S9. Immunofluorescent staining of Tuj1/GFAP in different groups at 1 day postinjury. Scar bar, 500 μm.

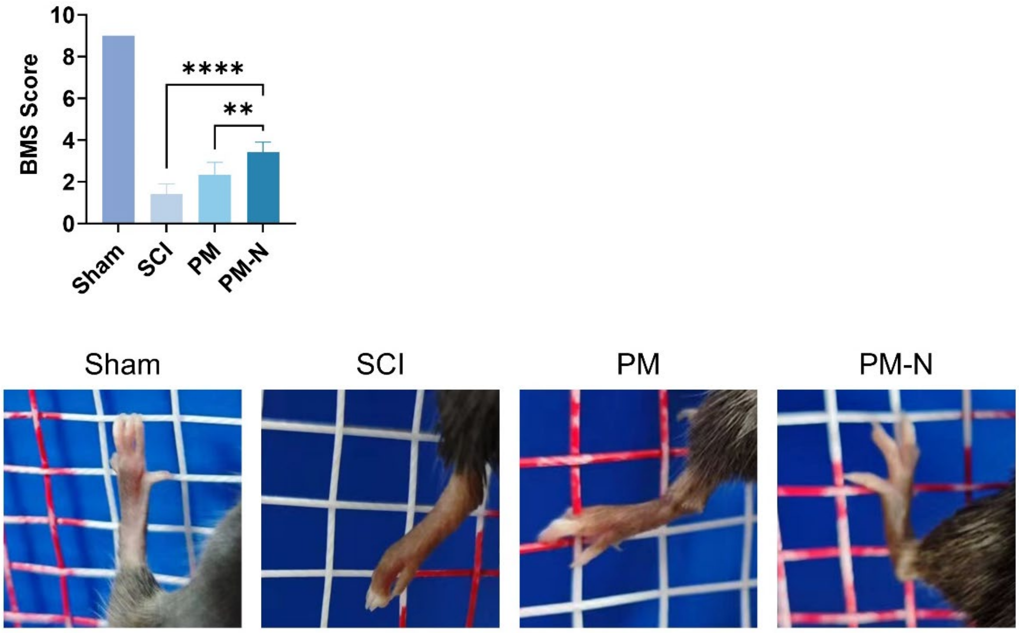

Figure S10. The BMS score and photograph of posterior limb at 28 days postinjury. n=6, **$p < 0.01$, ****$p < 0.0001$.

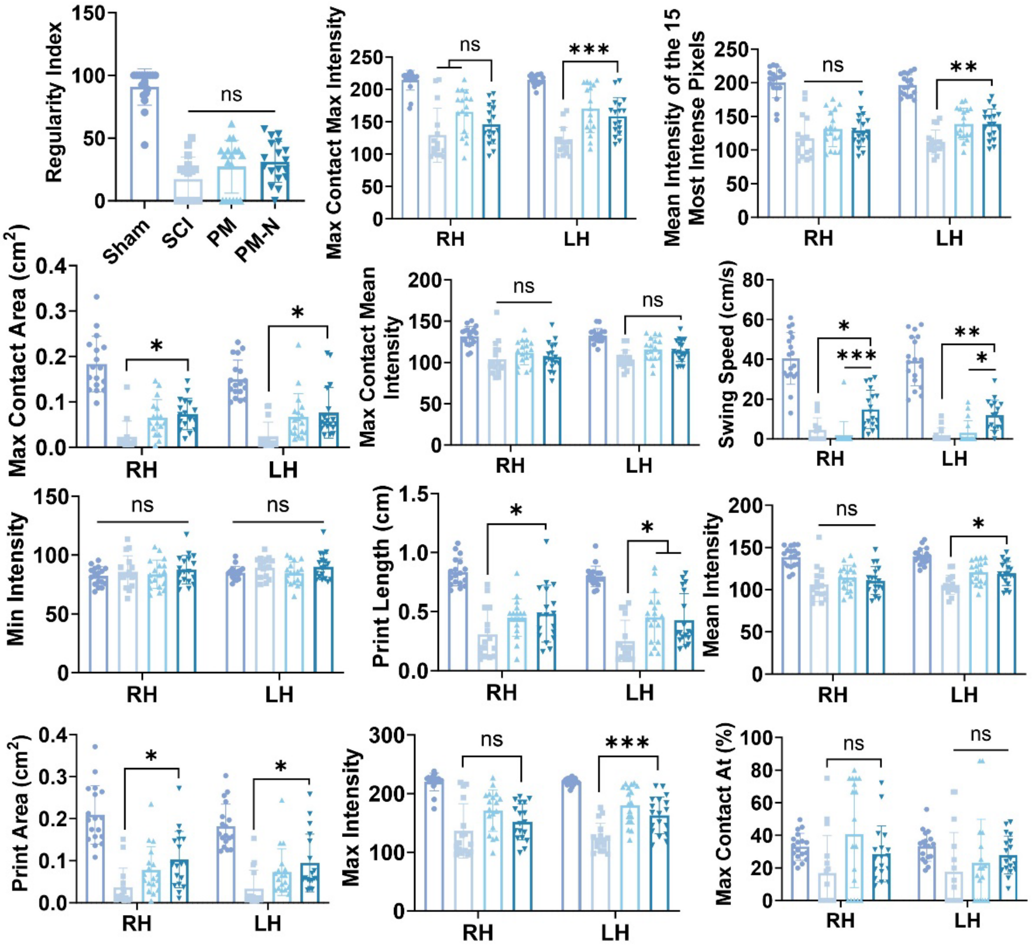

Figure S11. The analysis of CatWalk test at 28 days postinjury. n=18, $*p < 0.05$, $**p < 0.01$, $***p < 0.001$, ns, not significant.

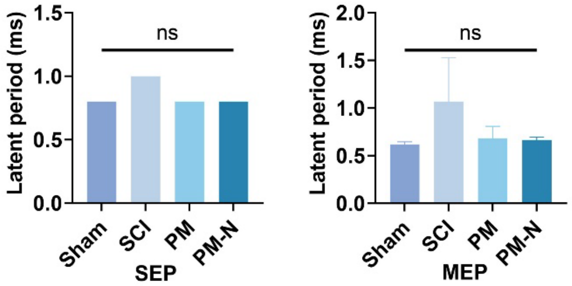

Figure S12. Latent period of SEP and MEP at 28 days postinjury. n=3, ns, not significant.

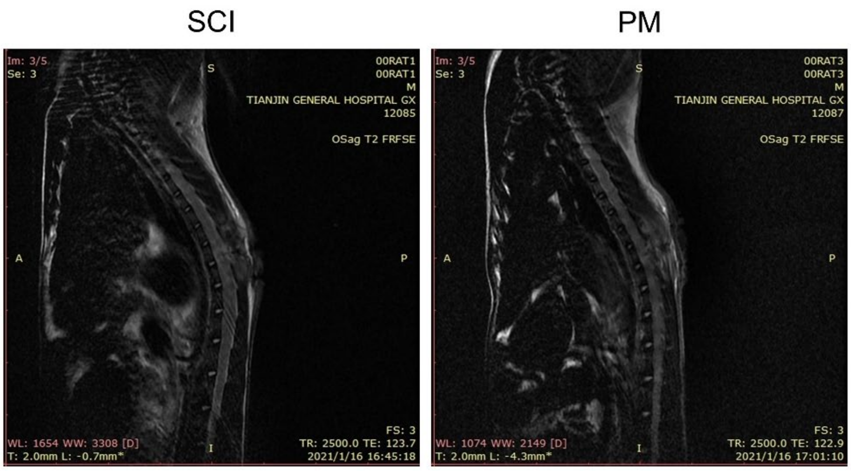

Figure S13. MRI of rats at 28 days postinjury.
